# The impact of tRNA modifications on translation in cancer: identifying novel therapeutic avenues

**DOI:** 10.1093/narcan/zcae012

**Published:** 2024-03-12

**Authors:** Ana M Añazco-Guenkova, Borja Miguel-López, Óscar Monteagudo-García, Raquel García-Vílchez, Sandra Blanco

**Affiliations:** Centro de Investigación del Cáncer and Instituto de Biología Molecular y Celular del Cáncer, Consejo Superior de Investigaciones Científicas (CSIC) - University of Salamanca, 37007 Salamanca, Spain; Instituto de Investigación Biomédica de Salamanca (IBSAL), Hospital Universitario de Salamanca, 37007 Salamanca, Spain; Centro de Investigación del Cáncer and Instituto de Biología Molecular y Celular del Cáncer, Consejo Superior de Investigaciones Científicas (CSIC) - University of Salamanca, 37007 Salamanca, Spain; Instituto de Investigación Biomédica de Salamanca (IBSAL), Hospital Universitario de Salamanca, 37007 Salamanca, Spain; Centro de Investigación del Cáncer and Instituto de Biología Molecular y Celular del Cáncer, Consejo Superior de Investigaciones Científicas (CSIC) - University of Salamanca, 37007 Salamanca, Spain; Instituto de Investigación Biomédica de Salamanca (IBSAL), Hospital Universitario de Salamanca, 37007 Salamanca, Spain; Centro de Investigación del Cáncer and Instituto de Biología Molecular y Celular del Cáncer, Consejo Superior de Investigaciones Científicas (CSIC) - University of Salamanca, 37007 Salamanca, Spain; Instituto de Investigación Biomédica de Salamanca (IBSAL), Hospital Universitario de Salamanca, 37007 Salamanca, Spain; Centro de Investigación del Cáncer and Instituto de Biología Molecular y Celular del Cáncer, Consejo Superior de Investigaciones Científicas (CSIC) - University of Salamanca, 37007 Salamanca, Spain; Instituto de Investigación Biomédica de Salamanca (IBSAL), Hospital Universitario de Salamanca, 37007 Salamanca, Spain

## Abstract

Recent advancements have illuminated the critical role of RNA modifications in post-transcriptional regulation, shaping the landscape of gene expression. This review explores how tRNA modifications emerge as critical players, fine-tuning functionalities that not only maintain the fidelity of protein synthesis but also dictate gene expression and translation profiles. Highlighting their dysregulation as a common denominator in various cancers, we systematically investigate the intersection of both cytosolic and mitochondrial tRNA modifications with cancer biology. These modifications impact key processes such as cell proliferation, tumorigenesis, migration, metastasis, bioenergetics and the modulation of the tumor immune microenvironment. The recurrence of altered tRNA modification patterns across different cancer types underscores their significance in cancer development, proposing them as potential biomarkers and as actionable targets to disrupt tumorigenic processes, offering new avenues for precision medicine in the battle against cancer.

## Graphical Abstract

**Graphical Abstract:**
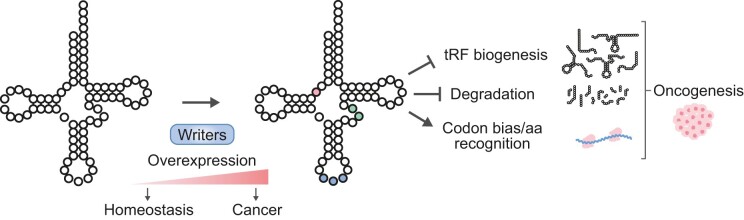

## Introduction

Protein synthesis is a highly preserved mechanism that involves the assembly of amino acids on ribosomes according to an messenger RNA (mRNA) template sequence. Translational control is crucial in modulating gene expression. Its significance extends to defining the proteome, ensuring homeostasis and governing cell proliferation, growth and development. Dysregulation of protein synthesis lies at the core of numerous diseases, underscoring the crucial need to understand the molecular foundations and intricate mechanisms of translational control (1).

Generally, protein synthesis is divided into three major steps: initiation, elongation and termination. The primary stage of regulation occurs during the recognition and decoding of the AUG start codon, managed by the methionyl-tRNA specialized for initiation (tRNAi^Met^). In eukaryotes, a scanning mechanism is employed, where the small (40S) ribosomal subunit, loaded with tRNAi^Met^ in a 43S pre-initiation complex (PIC), together with the eukaryotic initiation factors (eIFs) eiF2-TC, eIF3, eIF1 and eIF5, attaches to the mRNA near the 5′ end and scans the 5′-untranslated region (5′UTR) to identify an AUG (or less frequently a near-cognate AUG) codon. The mRNA is activated for 43S binding by the formation of the translation initiation complex eIF4F (eIF4A, eIF4B, eIF4E and eIF4E), which recognizes the mRNA’s m^7^G cap structure at the 5′ end and interacts with poly(A) tail-binding protein (PABP) at the 3′ end (2,3), forming the 48S ribosomal complex. Then, 60S ribosomes are recruited via eIF2-GTP activation, leaving the active 80S ribosomes to enter the extension phase (1). Then, protein elongation is produced by formation of a peptide bond of the aminoacyl-transfer RNAs complementary to the mRNA codon with the peptide attached to the ribosome P-site, followed by translocation of the ribosome. This process, promoted by distinct elongation factors and by the ribosome, is repeated until a termination codon enters the A-site, when termination factors bind the ribosome and promote the hydrolysis of the peptide (1). One of the main regulators of translation initiation, mechanistic target of rapamycin (mTOR), is a serine/threonine kinase situated downstream in the phosphoinositide 3-kinase (PI3K)/Akt signaling pathway, combining inputs from amino acid levels, extracellular stimuli as well as cellular oxygen and energy status. In normal conditions, mTOR inactivates by phosphorylation the initiation factor 4E-binding protein 1 (eIF4E-BP1), which releases eIF4E, allowing it to participate in the formation of the eIF4F complex (4). S6K mTOR-mediated activation, on the other hand, phosphorylates eIF4B on Ser422, to enhance the interaction with eIF3 and eukaryotic elongation factor-2 kinase (eEF2K) (5). However, mTOR inactivation, due to starvation or stress conditions, triggers cap-independent mRNA translation, which is IRES (internal ribosome entry site) dependent. This non-canonical protein translation is characterized by ribosomal direct recruitment of specific mRNAs that present discrete sequence elements in the 5′-UTR, and whose translation has been described to be induced under stressful situations (1,2).

Since translation is intricately linked to cell cycle advancement and cell growth, disruptions in protein synthesis are closely associated with tumorigenic processes. Therefore, inhibitors targeting the PI3K/Akt/mTOR pathway and, consequently, cap-dependent translation have evolved as therapeutic strategies for cancer treatment (6). Many mTOR inhibitors have been developed, but only a few of them have been approved to treat human cancer; the majority of mTOR inhibitors are currently undergoing evaluation in clinical trials (7). However, the PI3K/Akt/mTOR pathway is not the only translation regulatory mechanism dysregulated in cancer. Aberrant expression of various eIFs involved in distinct steps of the process, induction of cap-independent translation or even altered expression of ribosomal RNAs (rRNAs) and transfer RNAs (tRNAs) have been described during tumorigenesis [reviewed in detail in (8,9)].

Moreover, in recent years, post-transcriptional modifications have emerged as a critical regulatory level in numerous cellular processes (10–12). When focusing on protein synthesis, the tRNA epitranscriptomic modifications are key in regulation of this process. Within regulation of the epitranscriptomic, three key categories of proteins involved in RNA modifications stand out: writers, readers and erasers (WERs). Writers are responsible for adding modifications to RNA molecules, imparting diversity and complexity to genetic information (13). On the other hand, erasers play a crucial role in removing these modifications, allowing dynamic and precise regulation of gene expression (14). Meanwhile, readers are proteins that recognize these marks and trigger specific biological responses, including promoting the degradation of modified RNAs (15), boosting translation (16) or regulating mRNA splicing (17), among other roles. The interplay between these three types of proteins constitutes an intricate system that governs the function and plasticity of RNA molecules, playing a fundamental role in essential biological processes and exerting a crucial impact on diseases such as cancer.

The dynamic interplay among WERs is well understood in the context of mRNA, playing a crucial role in modulating RNA functionality and impacting a variety of biological processes and disease states, including cancer. When it comes to tRNA modifications, however, the scenario becomes more complex. While the roles of writers and erasers in introducing and removing modifications are clear, contributing significantly to tRNA structure and function, the concept of readers in tRNA biology is not as well defined. Over 110 modifications introduced by writers have been identified in tRNAs, some of which can be dynamically reversed; for instance, four demethylases known in humans, namely AlkB homolog 1 (ALKBH1) (18), ALKBH3 (19), ALKBH7 (20), and fat mass and obesity-associatedprotein (FTO) (21), can remove methylations. Despite these advances, the identification of readers that ‘interpret’ these modifications to mediate downstream effects in tRNAs remains elusive. This highlights a significant gap in our understanding of the dynamics and functional implications of tRNA modifications, underscoring the complexity and the need for further research in this area.

There are numerous mechanisms through which tRNA modifications can impact protein synthesis, yet the predominant mechanism involves boosting the production of proteins that drive tumor growth, characterized by specific codon usage patterns in their mRNAs (11,22,23). This mechanism, referred to as codon-biased translation, consists of the preferential translation of specific codons due to increased availability or stability of the tRNAs encoding the complementary anticodon, leading to differential translation of proteins enriched in those specific codons, initially identified in the regulation of stress responses (23), and proposed as modification-tunable transcripts (MoTTs) (24). This notion underscores how variations in wobble base tRNA modification levels can synergize with specific codon usage patterns within transcripts encoding essential stress response proteins where the cognate codons are significantly over-represented, thereby enhancing their translation efficiency, drawing a parallel to how codon bias in cancer can similarly regulate the translation of oncogenes or tumor suppressor genes. This process in cancer is supported by various studies revealing how modifications in the RNA, specifically within tRNAs, are used by oncogenic programs to stimulate proliferation and enhance resistance to chemotherapy (25). The key mechanism of codon-biased translation is exemplified by RNA-modifying proteins such as methyltransferase-1 (METTL1) and WD repeat domain 4 (WDR4), Elongator acetyltransferase complex subunit 1–6 (ELP1–6), cytosolic thiouridylase subunit 1–2 (CTU1–2) or AlkB homolog 8 (ALKBH8)-tRNA methyltransferase 112 (TRMT112), whose gene amplifications, increased expression or increased deposition of their modifications can contribute to the promotion of cancer proliferation (22).

Translational dysfunction is currently linked to over a hundred human diseases, particularly cancer (12). A comprehensive analysis of 34 writers in 55 types of cancer emphasized the recurrent dysregulation of the tRNA epitranscriptome in various cancers, pinpointing specific writers and modifications as potential drivers of differential codon-biased translation. Cancer databases such as The Cancer Genome Atlas (TCGA) and Cancer Cell Line Encyclopedia (CCLE) have exposed prevalent alterations in RNA-modifying proteins and tRNA expression, indicating the extensive engagement of codon-biased translation across various cancer types (22).

Here, we summarize the current state of knowledge concerning how tRNA-modifying proteins and their alteration regulate protein synthesis in the context of cancer progression. The latest findings give clearer insight into the molecular mechanisms by which, through an altered translation, these proteins are involved in critical functions of the tumor cells such as proliferation, migration or treatment resistance.

## Modifications of cytosolic tRNAs

Protein synthesis is a complex and multistep process based on the translation of the information encoded in mRNA into amino acids, which serve as the building blocks for proteins. This intricate process is mediated by tRNAs, functioning as carriers of amino acids during protein synthesis (26). Despite their non-coding nature, tRNAs play a crucial role in regulating gene expression and stand out as the RNA species most extensively modified (27).

All tRNA isotypes exhibit modifications along the cloverleaf structure, essential for regulating their function. However, the number of modifications varies among organisms, tissues and even among tRNA species. Eukaryotic cytosolic tRNAs typically display an average of 11–13 modifications within the 76 nucleotides that typically make up a tRNA (28).

Modifications in the tRNA functional domain, the anticodon loop, are complex and highly conserved, due to its critical role in ensuring translation fidelity (23,29,30). The wobble position (position 34), crucial for codon recognition specificity, exhibits the highest chemical variability, with >30 different identified modifications (27). Wobble modifications are indispensable for accurate protein translation, as they influence correct base pairing, codon usage, reading frame optimization and determination of the decoding capacity of a specific tRNA (29).

A noticeable example of the intricate functions of tRNA modifications is that of queuine, the queuosine (Q) nucleobase, located at position 34 in specific tRNAs (31). Derived from 7-deaza-guanine, queuine features an amino-methyl side chain and a cyclopentenediol moiety (32), and is catalyzed by the enzyme tRNA guanine transglycosylase (eTGT) (33). Q modification has a context-dependent impact on translational speed, favoring C-ending codons due to the higher stability of Q–U pairing compared with G–U pairing (34). Consequently, depletion of Q in human cells results in reduced translational speed at Q-decoded codons (35). Additionally, Q modification prevents misreading of C-ending glycine and cysteine codons without affecting U-ending codons, an evolutionarily advantageous feature under conditions of high Q modification. Furthermore, Q modification influences the activities of other writers, as observed with the Dnmt2 homologs Pmt1 from *Schizosaccharomyces pombe* and DnmA from *Dictyostelium discoideum*, which are stimulated by prior Q modification deposition (36), in mouse embryonic stem cells (mESCs), where Q modification of tRNA promotes Dnmt2-dependent m^5^C38 methylation, and in tissues, where loss of eTGT leads to reduced m^5^C38 levels (37).

Another example of anticodon modifications that ensure accurate protein translation are modifications at U34 in certain tRNAs in *Saccharomyces cerevisiae* and *Caenorhabditis elegans*. The most common modifications in this position are 5-methoxycarbonylmethyl (mcm^5^) and 5-carbamoylmethyl (ncm^5^) mediated by the six-subunit Elongator (Elp) complex (38); also in some tRNAs mcm^5^U34 addition is followed by a 2-thio group (s^2^) added by Uba4, Urm1, Ncs2 and Ncs6 enzymes (39). Loss of one of these modifications at U34 leads to slower decoding, ribosome pausing and widespread protein aggregation *in vivo* (40).

In essence, epitranscriptomic marks at the wobble position are essential for maintaining the degeneracy of the genetic code (41), and altered deposition of modifications at the wobble position can cause translation defects, such as mistranslation, paused translation of specific codons, speed or translation rate alteration (40,42,43).

In contrast, chemical modifications decorating the tRNA backbone, including the D- and T-loops, uphold molecule folding and stability (44). Although these modifications might not directly influence mRNA recognition by the tRNA, they play a crucial role in tRNA processing, thereby regulating protein synthesis in a more indirect manner. Altered deposition of these modifications is associated with tRNA degradation (26), cleavage and impaired processing (44,45). The relevance of these modifications in tRNA stability was first observed in yeast, where simple or combined aberrant deposition of non-essential tRNA modifications, such as *N*^7^-methylguanosine (m^7^G), *N*^4^-acetylcytidine (ac4C) and *N*^2^,*N*^2^-dimethylguanosine (m^2,2^G), result in rapid tRNA decay and activation of the general amino acid control (GAAC) stress pathway (46–50). Moreover, the lack of an extra guanine (G−1) at the 5′ end of tRNA^His^, catalyzed by the tRNA^His^ guanylyltransferase (Thg1p), leads to tRNA^His^ accumulation in the nucleus, with an additional 5-methylcytidine (m^5^C) modification, disrupting cell growth in yeast (51). Similarly, the absence of 2′-*O*-methylation at position 44 in tRNA^Ser(CGA)^, tRNA^Ser(UGA)^ and tRNA^Leu^, catalyzed by the tRNA methyltransferase 44 (Trm44), induces temperature sensitivity in these tRNAs, resulting in the loss of tRNA viability in yeast (52). These cases collectively suggest that modifications at different residues in the tRNA backbone impart distinct properties or introduce rigidity to the tRNA structure (53). Altered deposition of these modifications can lead to imbalanced tRNA stability (26), cleavage and impaired processing (44,45). For example, modifications outside the anticodon loop can lead to formation of tRNA-derived fragments (tRFs), which are small non-coding RNA molecules derived from tRNA cleavage (45,54–57). Interestingly, these tRFs have been described to actively regulate gene expression either in a manner similar to microRNA (miRNA) by associating with argonaute proteins (AGO) and the RNA-induced silencing complex (RISC) (58), or by directly inhibiting protein translation in specific situations (59). Consequently, tRNA modifications regulate both global and specific protein synthesis rates mainly by a distinct mechanism. First, anticodon modifications regulate amino acid recognition and codon usage, as well as biogenesis of tRFs, determining translation specificity, efficiency and speed. In addition, modifications outside the anticodon loop regulate protein synthesis by preserving their correct tertiary structure, stability and processing into tRFs with specific regulatory functions.

In summary, tRNA modifications are crucial for optimal translation, and any disruption in their regulation leads to disrupted protein synthesis. Impairment of this essential cellular process is intricately linked to the development of a spectrum of human pathologies, including cancer (12,60). Notably, the involvement of tRNA-modifying proteins (mainly writers and erasers) in various aspects of tumor development, progression and survival underscores the critical role of tRNA modifications in cancer biology (61). Consequently, a comprehensive understanding of the molecular mechanisms underlying tRNA modification and regulation is essential for both comprehension of the mechanisms driving tumor progression and the exploration of novel therapeutic approaches.

### *N* ^7^-Methylguanosine in cytosolic tRNAs

**Figure 1.**
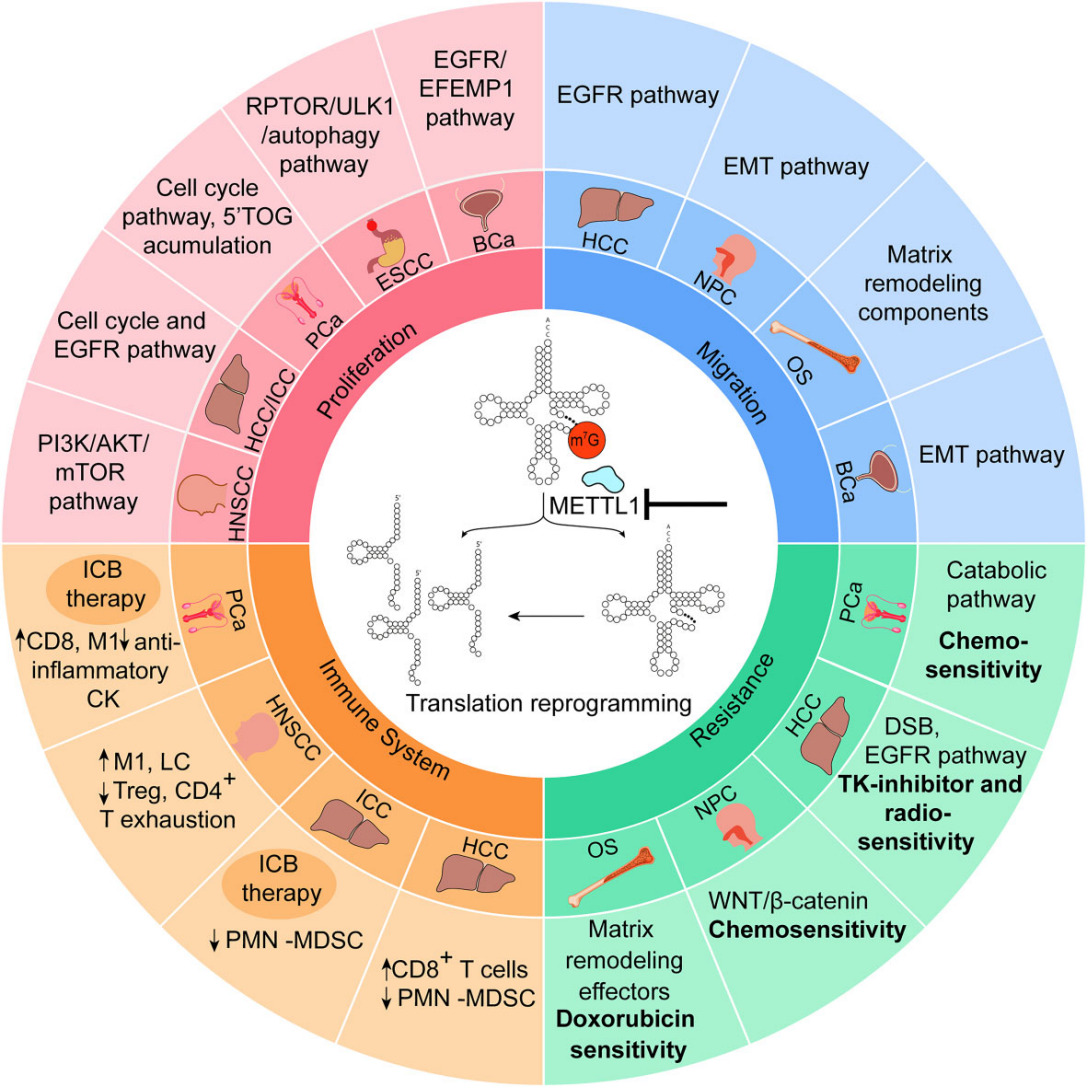
Overview of cancer-regulatory mechanisms affected by m^7^G methylation. The chart shows the cellular pathways affected in each tumor when METTL1 is deleted or silenced, via tRNA hypomethylation and/or tRNA fragments. Proliferation and migration are reduced in all cases. In all cancer types, METTL1 is overexpressed: hepatocellular carcinoma (HCC), intrahepatic cholangiocellular carcinoma (ICC), head and neck squamous cell carcinoma (HNSCC), prostate cancer (PCa), esophageal squamous cell carcinoma (ESCC), bladder cancer (BCa), nasopharyngeal carcinoma (NPC) and osteosarcoma (OS). ICB therapy: immune checkpoint blockade therapy.

One of the most prevalent modifications in cytosolic tRNAs is m^7^G, located at position 46 in the variable loop of a large subset of cytosolic tRNAs (62) (Figure 1). This modification has been detected in humans and in distinct prokaryote, archaea and eukaryote species. In addition to its prevalent presence on tRNAs, an internal m^7^G mark has also been identified in mRNAs (63), rRNAs (64) and precursor miRNAs (pre-miRNAs) (65), highlighting its biological significance.

In mammals, deposition of m^7^G is catalyzed by a heterodimeric protein complex comprising the catalytic subunit METTL1 and the regulatory unit WDR4, utilizing *S*-adenosylmethionine (SAM) as substrate (62,66,67). Despite not being essential for survival under normal culture conditions, mutations of the m^7^G yeast tRNA methyltransferase 82 (Trm82) result in rapid decay of hypomodified tRNAs and increased heat stress sensitivity (68). In mammals, m^7^G modification is indispensable for optimal protein translation and tRNA stability, and is required for mESC self-renewal and differentiation (69–72).

From a pathological perspective, METTL1 is overexpressed in a wide variety of cancers such as lung (LUAD) (73), prostate (PCa) (57,74), bladder (BCa) (75), head and neck squamous cell carcinoma (HNSCC) (76), breast cancer (BC), glioblastoma (GBM), melanoma, acute myelogenous leukemia (AML) (70), hepatocellular carcinoma (HCC) (77), intrahepatic cholangiocarcinoma (ICC) (71), osteosarcoma (78), nasopharyngeal carcinoma (NPC) (79) and esophageal squamous cellk carcinoma (ESCC) (72). Notably, METTL1’s molecular impact exhibits variability among different tumors, influencing key cancer hallmarks such as tumorigenesis, proliferation, stress response, the immune system and microenvironment cross-talk, and even regulation of the metastatic process (Figure 1).

When examining the role of METTL1 in various cancer types, the common theme emerges as a regulator of proliferation and tumorigenesis. Depletion of *METTL1* or its cofactor *WDR4* consistently results in decreased cell proliferation, colony formation and impaired tumorigenesis in LUAD (73), ICC (71), BCa (75), HCC (77), HNSCC (76), PCa (74) and ESCC (72). The impaired proliferation upon *METTL1* depletion consistently involved reduced translation of proliferation and cell cycle regulators, such as cyclin D3 and cyclin E1, as observed in lung cancer (73). In liver tumors, specifically ICC (71) and HCC (77), METTL1 also governed the translation of cyclin A2 and epidermal growth factor receptor (EGFR) proteins, consequently regulating the phosphorylation of the receptor downstream targets AKT, mTOR (71) and mitogen-activated protein kinase (MAPK) (77). This regulatory effect extends to bladder cancer, where METTL1 controls the translation of both EGFR and EGF-containing fibulin extracellular matrix protein 1 (EFEMP1), thereby regulating the activation of the PI3K/AKT and MAPK pathways (75). In HNSCC, the diminished phosphorylation of AKT and mTOR following *METTL1* depletion is mediated by the decreased translation of *PI3KCA*, accompanied by reduced translation of cyclin D1 and vimentin (76). In prostate cancer (PCa), the activation of the PI3K pathway induces increased *METTL1* expression, which in turn regulates a specific translational program concomitant with proliferation and survival (74). Thus, a common link between METTL1 and PI3K and MAPK pathways is described across several cancers, implying the cross-talk between key signaling pathways and post-transcriptional events resulting in higher proliferation and tumorigenesis.

Indeed, *METTL1* overexpression is consistently associated with poor patient prognosis. All these studies collectively established a common mechanism through which METTL1 regulates these phenotypes. Upon *METTL1* deletion, reduced translation of mRNAs with higher frequencies of m^7^G tRNA codons such as tRNA^Arg(TCT)^ are observed (70). This reduction is attributed to ribosome pausing at m^7^G-tRNA-decoded codons due to rapid decay or reduced stability of those tRNAs (70,71,73,75–77,80).

More recently, work in our group has unveiled an alternative mechanism through which METTL1 regulates tumor progression in PCa (74). Consistent with other tumors, METTL1 is overexpressed in PCa, leading to enhanced proliferation and tumorigenesis both *in vitro* and *in vivo*. Intriguingly, in PCa cell lines and mouse models, loss of m^7^G-tRNA methylation following *METTL1* depletion resulted in the biogenesis of a novel class of tRNA-derived fragments featuring a 5′-oligoguanine domain (5′TOG). These small non-coding RNAs induced a global inhibition of protein translation by sequestering canonical translation initiation factors, inhibiting the translation of cell cycle- and proliferation-related genes, while specifically reshaping the translational program to favor the translation of other transcripts (74).

Nevertheless, regardless of the specific molecular mechanism governing translation regulation, all the studies underscore the reliance of these phenotypes on the methyltransferase activity of METTL1. This is evident as the generation of catalytically dead mutants fails to elicit these effects (70,71,73,74,76,77).

Interestingly, inhibition of METTL1 in PCa not only decreased cell proliferation but also induced catabolic and stress response mechanisms through increased translation of interferon (IFN) signaling pathway effector genes. These conditions triggered the activation of IFN signaling in cancer cells, thereby increasing immune infiltration of cytotoxic immune cells in the tumor microenvironment (TME) (74). Additionally, the absence of *METTL1* in PCa disrupted autophagy resolution and increased proteostatic stress, resulting in the accumulation of reactive oxygen species (ROS) and DNA damage, and heightened sensitivity to chemotherapeutic agents such as docetaxel and etoposide (57). A similar inverse correlation between *METTL1* expression and autophagy induction was observed in ESCC. This connection was mediated by the METTL1-dependent translation of regulatory associated protein of mTOR complex 1 (*RPTOR*), an activator of the mTOR pathway and a negative regulator of autophagy (72).

The increased cytotoxicity and therapeutic resistance of tumor cells upon METTL1 inhibition are not exclusive to PCa and ESCC. Indeed, although the molecular effectors vary across cancer types, and some mechanisms are not yet totally uncovered, METTL1-dependent m^7^G tRNA modification is essential for modulating cancer cell responses to stress and therapeutic challenges, being crucial for resistance acquisition. For example in HCC, METTL1 expression promotes tumor resistance to both radiotherapy (80) and the tyrosine kinase inhibitor levatinib (81), enhancing the translation of the DNA double-strand break (DSB) repair enzyme DNA ligase IV or components of the EGFR pathway, respectively. In NPC, METTL1 overexpression induces tumorigenesis, chemoresistance and epithelial–mesenchymal transition (EMT) by up-regulating the Wingless and Int-1/β-catenin (WNT/β-catenin) signaling pathway through increased WNT3A protein translation (79). Rescue of WNT3A expression in *METTL1* knockout (KO) NPC cells bypasses the enhanced sensitivity to docetaxel and cisplatin observed upon methyltransferase depletion (79). Moreover, increased doxorubicin resistance has been linked with higher METTL1 levels in osteosarcoma, coupled with up-regulated migration, invasion and proliferation mediated by increased translation of the extracellular matrix organizer LOXL2 (78).

Another important challenge to overcome when facing cancer is tumor metastasis, and METTL1 appears to play a role in this critical scenario. As mentioned earlier, the METTL1-dependent translational program regulates the migration and invasion capacity of liver and bladder tumor cells via EGFR up-regulation (71,75,77). This mechanism may contribute to the metastatic potential of HCC cells observed upon radiofrequency-induced METTL1 expression (82). Moreover, m^7^G-dependent translation of either the EMT (79) or extracellular matrix-remodeling components (78) regulates migration of nasopharyngeal and osteosarcoma cells, respectively. In addition, the deletion of *Mettl1* in an HNSCC mouse model significantly reduced lymph node metastasis in a PI3K/AKT-dependent manner (76).

While METTL1 primarily intervenes in translation programs driven by m^7^G-tRNA mechanisms, Pandolfini *et al.* described a novel metastasis regulatory mechanism mediated by pre-miRNA methylation, particularly those from the let-7 family. METTL1-dependent m^7^G modification was shown to be essential for both pre-miRNA processing and mature miRNA function, which in turn mediated stability of key EMT and migration transcripts, consisting of specific regulation of cell migration (65).

Thus, the m^7^G mark not only governs intracellular processes and stress responses in tumor cells but also plays a crucial role in regulating their cross-talk with other cells, such as the immune system. In fact, several studies highlight METTL1’s regulatory role of the immune landscape in several tumor types towards a more anti-tumoral profile. In an HNSCC mouse model, *Mettl1* depletion led to an increase in the infiltration of M1-like macrophages and Langerhans cells (LCs), and a reduced presence of regulatory T cells and CD4^+^ exhaustion cells within the tumor (76). This switch was associated with the inhibition of the stromal and epithelial cell receptor–ligand pairs of the interleukin family Il1b–Il1r2 and Adrb2 (76). This immune–tumor cell cross-talk modulatory function was also observed in PCa, where the loss of the m^7^G mark altered cytokine secretion via translational activation of IFN pathway genes (74). Consequently, increased secretion of pro-inflammatory cytokines occurred both *in vitro* and *in vivo*, polarizing immune cells towards a more cytotoxic phenotype. PCa is characterized as a ‘cold tumor’ due to its extensive infiltration of myeloid pro-tumoral cells, leading to reduced efficacy of immune checkpoint blockade (ICB) therapy. Strategies aimed at repolarizing it toward a more cytotoxic phenotype could significantly enhance the effectiveness of ICB inhibitors. Notably, the deletion of *Mettl1* in mouse models of PCa induced the desired shift, increasing infiltration of M1-like macrophages and CD8 T cells, and ultimately improving the efficacy of anti-PD1 therapy (74).

A similar perspective is seen in ICC (83) and HCC (84) tumors, where METTL1 overexpression is correlated with an immunosuppressive environment caused by accumulation of polymorphonuclear myeloid-derived suppressor cells (PMN-MDSCs). This process is mediated by METTL1-induced translation of distinct key cytokines; human interleukin 8 (IL8) and mouse C-X-C motif chemokine 5 (Cxcl5) in ICC (83), and transforming growth factor-β2 (TGF-β2) in HCC tumors (84). Moreover, increased efficacy of anti-PD-1 therapy was observed in an ICC pre-clinical mouse model following co-blockade of METTL1 and its downstream chemokine pathway (83).

In summary, METTL1 emerges as a versatile contributor to cancer biology by intricately regulating protein translation. While the molecular mechanisms vary across distinct tumors due to tissue-specific translational programs, the global impact of METTL1 expression is consistently maintained. The METTL1-dependent m^7^G status significantly influences tumor proliferation, therapy response, metastasis and the immune microenvironment. This highlights METTL1’s potential as a therapeutic target and positions it as a key player in comprehending the dynamics of cancer progression.

### Other cytosolic tRNA modifications in proliferation and tumorigenesis control

**Figure 2.**
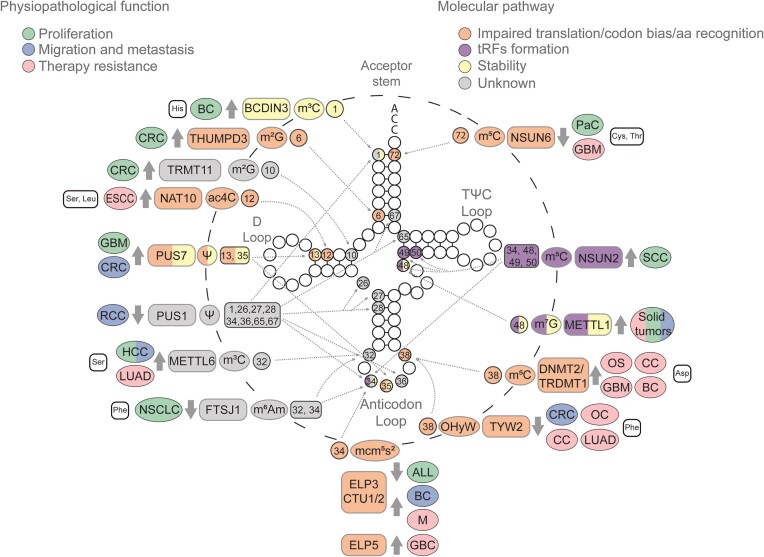
Cytosolic tRNA modifications impacting tumoral processes. Epitranscriptomic marks deposited in cytosolic tRNAs have been implicated in various cancerous processes, including proliferation (green), migration and metastasis (blue), and therapy resistance (pink). These consequences arise from diverse molecular alterations such as impaired translation or codon bias (orange), the biogenesis of tRFs (purple), tRNA stability (yellow) or a still unknown mechanism (gray). The illustration progresses from the outermost to the innermost layers, detailing the tRNA isotype that undergoes modification—if no tRNA isotype is indicated, several tRNA isotypes are involved—; the associated tumor type; the enzyme responsible for each mark, and whether it is up- or down-regulated (indicated by gray thick arrows); the mark; and the position of the mark. Solid tumors encompass all the solid tumors specified in this figure. Modifications located on the anticodon loop, D arm and acceptor stem are highlighted to indicate their involvement in amino acid recognition, codon usage and tRNA stability. Meanwhile, those modifications situated at the variable loop are indicated as being involved in tRF biogenesis. Glioblastoma (GBM), ovarian carcinoma (OC), cervical carcinoma (CC), osteosarcoma (OS), esophageal carcinoma (ESCC), lung adenocarcinoma (LUAD), melanoma (M), gallbladder cancer (GBC), squamous cell carcinoma (SCC), acute lymphoblastic leukemia (ALL), non-small cell lung cancer (NSCLC), colorectal cancer (CRC), hepatocellular carcinoma (HCC), pancreatic cancer (PaC), breast cancer (BC) and renal cell carcinoma (RCC).

It is well established that tRNA modifications play a critical role in sustaining key characteristics of cancer cells, including heightened proliferation, enhanced metastatic capabilities and the survival of cancer stem cells (CSCs) (85). Cancer cells, renowned for their elevated global protein synthesis rates compared with non-cancerous cells, highlight the integral role of tRNA modifications in reshaping cancer-associated phenotypes. The intricate link between increased cell proliferation and translational reprogramming of the tumor genome emphasizes the significance of tRNA modifications in diverse cancer types (8,86). Notably, an escalating number of alterations in expression of enzymes involved in tRNA modification processes are shown to contribute to cancer (87) (Figure 2).

RNA 5-methylcytosine (m^5^C), similar to m^7^G, is a prevalent post-transcriptional modification, orchestrated by members of the highly conserved NOL1/NOP2/Sun (NSUN) domain-containing family (27). NOP2/Sun RNA methyltransferase 2 (NSUN2) specifically catalyzes the m^5^C modification at positions 34 and 48 of intron-containing tRNA^Leu^ (CAA) precursors, and at positions 48, 49 and 50 of most tRNAs (56,88,89). In squamous cell carcinoma (SCC), NSUN2 plays a dual role by supporting tumor growth and ensuring cell survival during stress (90). Mechanistically, NSUN2-mediated m^5^C methylation protects tRNAs from cleavage, preventing the accumulation of non-coding 5′ tRFs (56). This mechanism facilitates global protein synthesis repression, reducing the expression of differentiation-related genes but surprisingly favoring the translation of key stress response genes (90). Thus, under external stress conditions, such as chemotherapy, when NSUN2 activity is impaired, tRNA cleavage into tRFs is enhanced, subsequently sensitizing specifically CSCs to stress (90). Indeed, depleting *Nsun2* led to decreased growth of SCC cells in mice, and increased sensitivity to chemotherapeutic agents (90). These findings anticipated that the combined use of methylase inhibitors with chemotherapy agents may hold promise as a potential therapy for eradicating CSCs.

Another important NSUN family member is NSUN6. NSUN6 catalyzes methylation at C72 of tRNA^Cys^ and tRNA^Thr^ isoacceptors (91). Recent studies have also described its capacity to methylate C48, C49 and C50 (92), but these findings remain controversial. In pancreatic cancer, *NSUN6* levels are reduced (93). The regulatory role of NSUN6 in cellular proliferation was confirmed through *in vitro* functional assays with pancreatic cancer cell lines and *in vivo* with mouse xenograft models, where *NSUN6* overexpression suppressed tumor growth and up-regulated cyclin-dependent kinase 10 (CDK10) at the transcriptional level (93). This suggested that NSUN6 may influence pancreatic cancer proliferation by regulating CDK10 expression involved in mitotic spindle assembly and mitotic nuclear division. Indeed, processes associated with cell proliferation, such as the cell cycle and G2/M checkpoint, were also found to be enriched in the group of pancreatic cancer patients with lower expression of *NSUN6*. However, whether NSUN6 influences proliferation-related genes post-transcriptionally is still not clear and needs further investigation.

Pseudouridine (Ψ), an isomer of uridine, competes with methylation to be the most predominant RNA modification (94). Conversion of uridine into Ψ is catalyzed by RNA-dependent pseudouridine synthases [dyskerin pseudouridine synthase 1 (DKC1) as the catalytic unit] and stand-alone pseudouridine synthase (PUS) (60). Elevated levels of Ψ deposition have long been associated with an increased cancer risk, as its high secretion in urine was identified in cancer patients (95,96). In glioblastoma multiforme (GBM), elevated expression of PUS7 has been observed (97). This protein modifies positions 13 and 35 of tRNAs (98). Recent findings demonstrated that suppressing *PUS7* drastically reduced the self-renewal of glioblastoma stem cells (GSCs), leading to tumor progression inhibition in mice (97). In terms of mechanism, the study revealed that PUS7 altered GSC growth by a codon-specific translational control of key GSC regulators. In particular, PUS7-mediated tRNA modification modulated the translation of tyrosine kinase 2 (TYK2)–signal transducer and activator of transcription 1 (STAT1), shedding light on its crucial role in GBM development. In addition, PUS7 dysregulation was also implicated in myelodysplastic syndromes (MDSs), a group of cancers in which blood cells do not mature in the bone marrow (99). In this study, the authors demonstrated that PUS7, operating in hematopoietic stem cells and ESCs, mediates the formation of Ψ-containing tRNA fragments. These fragments had the capability to repress global protein synthesis by bindingv to and obstructing the translation initiation complex, thereby favoring the translation of specific transcripts concerned with the tumorigenic process (99,100). Notably, this mechanism shed light on novel translational regulation processes, encompassing the formation of small non-coding RNAs derived from tRNA fragments.

5-Methoxycarbonylmethyl-2-thioylation (mcm^5^s^2^) is a highly conserved tRNA modification deposited at the wobble U34 in 11 cytosolic tRNA species through an enzymatic cascade, where ELP3 is the catalytic subunit (101,102). This modification significantly influences translation fidelity by distinguishing target tRNAs (102). Consequently, ELP3 deficiency was shown to activate a p53-dependent checkpoint, accompanied by activating transcription factor 4 (ATF4) overactivation and heightened protein synthesis. Thereby, *ELP3* loss promotes p53-mutated leukemia/lymphoma, and joint inactivation of p53 and ELP3 enhances tumorigenesis (103). While exploring the co-activation of p53 and ATF4 due to *ELP3* deletion requires further investigation, the results draw parallels with the response seen in yeast lacking U34-modifying enzymes, which resembles the effects of amino acid deprivation (104,105). In addition, different studies have indicated the elongator complex's importance in tumor development across different cancer types, including breast, intestinal and melanoma, mainly by modulating protein synthesis through a codon bias process (101,106,107).

In non-small cell lung cancer (NSCLC), a significant reduction of 2′-*O*-methyladenosine (m^6^Am) levels has been observed in tumor tissues (108). Reduced levels of m^6^Am are correlated with decreased FTSJ RNA 2′-*O*-methyltransferase 1 (FTSJ1) expression in NSCLC tissues and cells, suggesting that FTSJ1 mediates its deposition. The study also showed that increasing FTSJ1 expression in NSCLC inhibited cell proliferation and migration *in vitro* and *in vivo*, suggesting a tumor-suppressive role for m^6^Am deposition (108). Mechanistically, FTSJ1 methylated the 2′-*O*-ribose of nucleotides at positions 32 and 34 of tRNA^Phe^ (109). Its inhibition increased expression of DNA damage-regulated autophagy modulator 1 (DRAM1) at the transcriptional level, a gene crucial in TP53-mediated autophagy and apoptosis (108). FTSJ1 also plays a crucial role in X-linked intellectual disability (110). In X-linked intellectual disability studies, ribosome profiling analysis revealed a significant reduction in translation efficiency for genes crucial in supporting synaptic organization and function, supporting a role as well in controlling protein translation efficiency (110).

Recently, two poorly characterized putative methyltransferases, tRNA methyltransferase 11 homolog (TRMT11) and THUMP domain-containing 3 (THUMPD3), were identified as active *N*^2^-methylguanosine (m^2^G) modifiers targeting positions 10 and 6 of tRNAs, respectively (111). In colon cancer (CRC), while the absence of these m^2^G tRNA methyltransferases individually showed minimal impact on cell proliferation or mRNA translation, their concurrent deficiency significantly diminished both cell growth and protein synthesis, underscoring their combined importance in eukaryotic cells (111). The *TRMT11/THUMPD3* double KO revealed marked reduction in monosome and polysome formation, indicative of impaired translation. Furthermore, this study highlighted that both THUMPD3 and TRMT11 are indispensable for m^2^G introduction in tRNAs, emphasizing their crucial role in optimal protein synthesis and cell proliferation (111). Both proteins interact with the allosteric regulator of methyltransferases, tRNA methyltransferase activator subunit 11-2 (TRMT112) (112,113), known for its interaction with other enzymes that also has a profound impact in tumor growth and translation alteration, such as ALKBH8 or TRMT9A, TRMT9B and methyltransferase 5 (METTL5) (114–116), emphasizing the intricate network of interactions crucial for cellular processes such as protein synthesis and proliferation.

A diverse range of RNA methylations are catalyzed by the METTLs family (87). Specifically, METTL6 catalyzes 3-methylcytidine (m^3^C) formation at C32 in certain tRNA^Ser^ isoacceptors (117). *Mettl6* absence impacts mRNA levels and translation, encompassing genes related to proliferation, signaling, growth control and proteostasis, potentially inducing cellular dysfunction and affecting oncogenic programs. Concomitantly, *METTL6* deficiency led to reduced growth rates and self-renewal in mESCs and HepG2 cells, highlighting METTL6’s essential role in ESCs and transformed cells, and linking m^3^C tRNA modification to proliferation regulation. Indeed, in HCC, this protein is crucial for tumor cell growth *in vitro* and in a mouse xenograft model (117). Importantly, patients with low METTL6 levels in HCC exhibit increased survival rates, aligning with previous findings in human breast cancer cells (118), indicating a context-dependent role for METTL6 in cell growth across various cell types (119). These results emphasize the multifaceted impact of METTL6 on cellular processes and its potential significance as a therapeutic target.

Bicoid-interacting protein 3 (BCDIN3) is a methylase with dual activity towards pre-miRNAs (120) and the cytoplasmic tRNA^His^ (121). BCDIN3D-mediated 5′-monomethylation protects the tRNA^His^ from degradation by functioning as a capping enzyme (121). BCDIN3 is found to be overexpressed in breast cancer (122); however, the role of BCDIN3D-mediated tRNA 5′-monomethylation in the tumorigenic characteristics of breast cancer cells is not yet fully understood.

These findings highlight that cancer cells leverage tRNA modification not only to enhance translation rates but, more significantly, to alter codon bias usage, promoting the translation of transcripts that fuel proliferation and tumorigenesis.

### Cytosolic tRNA modifications in migration and metastasis

When Hannahan and Weinberg established the first six hallmarks of cancer, invasion and metastasis were identified as crucial mechanisms for tumor progression (123,124). Currently, with 14 recognized hallmarks, metastasis remains clinically relevant and a leading cause of cancer-related deaths (123,125). Recent discoveries have implicated post-transcriptional regulation including altered regulation of tRNA-modifying enzymes in metastatic processes across various cancer types.

In breast cancer, changes in mcm^5^s^2^ modification are associated with sustaining metastasis (101). ELP3 and CTU1/2, involved in mcm^5^s^2^ modification, are up-regulated in breast cancer (101). *In vitro* depletion of these genes resulted in reduced migratory potential of invasive breast cancer cells. Mechanistically, mcm^5^s^2^ modification enhanced *DEK* translation, promoting lymphoid enhancer-binding factor-1 (LEF1) translation through its IRES sequence, which resulted in a pro-invasive phenotype (101).

The m^3^C methyltransferase METTL6 exhibits elevated expression in HCC (126). Its heightened expression is correlated with an unfavorable prognosis and is linked to increased metastasis (126). Mechanistically, *METTL6* KO significantly reduced the transcriptional expression of cell adhesion-related genes, including integrin α-1/β-1 (ITGA1), claudin-14 (CLDN14) and spondin1 (SPON1), as revealed by RNA sequencing. Chromatin immunoprecipitation sequencing (ChIP-seq) results showed no notable differences in enhancer activities, suggesting that METTL6 potentially modulated target genes post-transcriptionally (126). Consequently, through these post-transcriptional alterations, *METTL6* depletion inhibited cell migration, invasion and adhesion of HCC cells (126).

In CRC, the epigenetic inactivation of tRNA-yW synthesizing protein 2 (TYW2) is linked to a poor prognosis in patients and increased metastatic capacity in CRC cells. TYW2, together with six enzymes, TYW1–TYW5 and tRNA methyltransferase 5 (TRMT5), sequentially catalyze the OHyW (hydroxywybutosine) modification at position 37 of tRNA^Phe^. Hypermethylation of the *TYW2* promoter and its consequent silencing result in hypomodification of guanine 37 (G37). The absence of this modification increases ribosome frameshifts, affecting protein translation of key cancer-related proteins such as roundabout guidance receptor 1 (ROBO1) (127), the loss of which inhibits cellular migration and EMT (128).

Altered pseudouridine deposition has also been associated with metastasis, particularly through PUS1 and PUS7 dysregulation (129,130). PUS7 is found to be overexpressed in CRC (129). Its oncogenic role and therapeutic potential were demonstrated by functional studies where silencing repressed CRC cell metastasis, while its up-regulation promoted it. Mechanistically, albeit independently of PUS7 catalytic activity, LIM and SH3 domain protein 1 (LASP1) overexpression or heat shock protein 90 (HSP90) inhibition rescued the phenotype of PUS7 overexpression, suggesting a possible post-transcriptional regulation of *LASP1* and *HSP90* expression (129). PUS1 pseudouridylates tRNAs at positions 1, 26, 27, 28, 34, 36, 65 and 67 (131). High PUS1 expression is closely associated with renal cell carcinoma (RCC) (130). In fact, up-regulation of PUS1 enhances RCC cancer cell viability, migration, invasion and colony formation ability, while decreased PUS1 expression exerts the opposite effects on RCC cells (130). However, the mechanism involving tRNA pseudouridylation and both cancer types is not yet fully understood (129,130).

### Cytosolic tRNA modifications shaping the immune microenvironment compartment

These findings collectively underscore the involvement of tRNA modifications in the initiation and advancement of cancers, influencing critical aspects such as proliferation and migration. However, cancer, like other pathologies, is not solely dictated by the molecular processes occurring within tumor cells; it significantly relies on the microenvironment, particularly the immune cells in its vicinity. Recent investigations have unveiled that the critical role of epitranscriptomic modifications in mRNA altering translation and protein synthesis impacts the function and infiltration of immune cells (132). This highlights the multifaceted influence of RNA modifications on the complex interplay between cancer cells and the immune microenvironment, influencing the tumor's ability to evade immune surveillance and mount effective anti-tumor responses or being involved in immune cell activation.

Indeed, recent advances have also highlighted that tRNA modifications, through post-transcriptional and translational control, can influence the activity of immune cells and the tumor–immune cross-talk. For example, wybutosine and 2-methylthio-*N*^6^-threonylcarbamoyladenosine (ms^2^t^6^A), that occur in the anticodon loop of tRNAs, have been shown to be involved in the activation of T lymphocytes, through alteration of the ribosome frameshifting (133).

To effectively combat pathogens, CD4 T cells must rapidly respond and reprogram their molecular machinery. Methylation of 1-methyladenosine at position 58 (m^1^A58) of most tRNAs has emerged as a crucial mechanism, facilitating rapid protein synthesis and propelling activated T cells into quick mitosis and proliferation (134). Using genetic mouse models and RNA sequencing, it was demonstrated that the proteins responsible for this modification, tRNA methyltransferase 6 (TRMT6) and the catalytic subunit tRNA methyltransferase 61A (TRMT61A), rapidly increased their expression upon T-cell activation. An increase in m^1^A58 deposition was shown to support the translation needs for proteins crucial at the pre-cell cycling stage of T-cell activation (134). These results unveiled a potential therapeutic target for T-cell-related inflammatory diseases and cancer immunotherapy.

ELP3 is also involved in CD4 T activation, as this deficiency in naive CD4 T cells delays their entry into the cell cycle upon activation (135). Indeed, *in vivo* immunization models of *Elp3*-deficient T cells demonstrated reduced expansion, leading to impaired T follicular helper (TFH) responses. Further transcriptomic analyses revealed a progressive overactivation of the stress-responsive transcription factor ATF4 in *ELP3*-deficient T cells; however, the molecular mechanism underlying this alteration in gene expression is still not fully understood (135). Whether *ELP3* deficiency can influence tumor progression by impairing T-cell activation remains undetermined.

Other important cellular subsets in TME are macrophages. In this context, ELP3 plays a crucial role in regulating macrophage polarization, limiting M1 and promoting M2 phenotypes. In myeloid cells, *ELP3* expression is down-regulated by classical M1 signals, limiting pro-inflammatory cytokine production via forkhead box O1 (FOXO1) phosphorylation (136). Conversely, alternative M2 signals elevate *ELP3* expression through a PI3K- and STAT6-dependent pathway. ELP3’s impact extends to WNT-driven tumor development in the intestine by sustaining a pool of M2-like macrophages within tumors (136). Mechanistically, ELP3 regulates the translation of key proteins, including mitochondrial ribosome subunits, and activates mTOR complex 2 (mTORC2) by promoting the translation of its activator, guanine nucleotide exchange factor B (RIC8B).

The comprehensive understanding of RNA modifications opens up avenues for potential clinical interventions, particularly in the intersection of immunotherapy and tRNA modifiers. The prospect of targeting tRNA modifiers in conjunction with immunotherapy holds promise for developing more effective and tailored treatments, advancing our capabilities in the ongoing fight against cancer.

### Cytosolic tRNA modifications in therapy resistance

Significant strides have been made in cancer therapy; however, drug resistance remains a formidable challenge in reducing cancer-related fatalities. Initially, the approach to overcome drug resistance involved target therapy and combination treatments, which proved effective in some cancers but fell short in fully addressing drug resistance (137,138). Novel strategies are imperative to tackle this issue, leading to the exploration of genes associated with therapy resistance. The emerging field of epitranscriptomics has primarily focused on mRNA modifications in relation to treatment resistance in cancer (139). Nevertheless, it is essential to recognize that mRNA is not the sole RNA implicated in drug resistance, and recent advances in cancer epitranscriptomics have indicated tRNA-modifying enzymes as novel players.

Various enzymes involved in modifying wobble uridine 34 in tRNAs have been linked to resistance against targeted therapies (106,140,141). ELP3 and CTU1/2 were shown to enhance glycolysis in melanoma cells by up-regulating hypoxia-inducible factor 1 subunit alpha (HIF1A) translation through codon-dependent regulation, contributing to their resistance to RAF inhibitors such as vemurafenib or dabrafenib (106,142). In other study, by performing a genome-wide CRISPR screening, the authors identified another U34 tRNA-modifying enzyme, ELP5, as an important regulator in drug resistance in gallbladder cancer (GBC) (141). Loss of *ELP5* impaired the stability of the elongator complex, hindering U34 modification. Consequently, hypomodification of U34 resulted in down-regulated expression of heterogeneous nuclear ribonucleoprotein Q (hnRNPQ), leading to a decrease in *TP53* IRES-dependent translation, ultimately reducing gemcitabine-induced apoptosis and decreasing gemcitabine sensitivity in GBC cells (141).

Beyond 34, position 37 at the anticodon loop is also crucial for drug resistance. TYW1 and TYW2 sequentially catalyze the formation of 4-demethylwyosine (imG-14) followed by OHyW at position G37 of tRNA^Phe^ (143); thus, in cell lines lacking *TYW2*, a shift towards the predominance of imG-14 modification in G37 occurs (144). Interestingly, Pan and colleagues found that cells with an accumulation of imG-14 were more resistant to taxol therapies, commonly used in cancer treatment, highlighting that novel therapies engineered to reduce imG-14 modification could re-sensitize cells to taxol treatment, overcoming drug resistance (144).

Additionally, alterations in DNA methyltransferase 2/tRNA aspartic acid methyltransferase 1 (DNMT2/TRDMT1) have been linked to cancer and drug resistance (145–147). DNMT2 is involved in m^5^C methylation of tRNA^Asp^ at position C38 (148). This methylation plays a role in the discrimination of the near-cognate codons, thereby ensuring accurate polypeptide synthesis (149). Its loss has been shown to increase sensitivity to DSBs, and thus its inhibition could be a potential target together with DNA damage repair (DDR) inhibitors (146,147). Indeed, in GBM, loss of *DNMT2* reduces apoptosis and senescence while increasing necrosis (146). Furthermore, its loss in osteosarcoma cells increases sensitivity to poly(ADP-ribose) polymerase (PARP) inhibitors (147). Mechanistically, in osteosarcoma cells, DNMT2 is recruited to DSBs to induce mRNA m^5^C at DNA damage sites, thereby recruiting RAD51 and RAD52, which finally promote DDR by homologous recombination (HR) (147). In other cancer types, *DNMT2* KO affects the unfolded protein response, sensitizing cells to endoplasmic reticulum stress (ERS)-induced apoptosis after doxorubicin treatment (145). Whether DNMT2-mediated tRNA methylation and translation control play a role in activation of DDR or ERS pathways has not been addressed.

Another tRNA modification implicated in drug resistance is ac4C. This modification is introduced by *N*-acetyltransferase 10 (NAT10) and tRNA acetyltransferase (TAN1) at position 12 of tRNA^Ser^ and tRNA^Leu^ (150). NAT10 is up-regulated in several cancers and is associated with a poor prognosis (151). In esophageal carcinoma (ESCA), its up-regulation promotes tumorigenesis through the EGFR pathway. In addition, *NAT10* depletion increases ESCA sensitivity to gefitinib treatment (152). While mechanistically the presence of ac4C in tRNAs has been shown to enhance the fidelity of protein translation (151), the connection between increased fidelity of translation and tumorigenesis and gefitinib responses mediated by increased NAT10 expression need future investigations.

m^5^C in tRNA has also been associated with drug resistance in cancer. Notably, high expression of NSUN6 directly correlates with decreased temozolomide resistance in GBM (153). Mechanistically, NSUN6-mediated tRNA methylation regulates transcriptional pauses, leading to the accumulation of negative elongation factor complex member B (NELFB) and general transcription factor complexes (*POLR2A*, *TBP*, *TFIIA* and *TFIIE*) on the PIC at the TATA-binding site. This intricate regulation influences the translation machinery in the GBM response to alkylating agents.

The deep understanding of tRNA modifications offers exciting prospects for clinical interventions, particularly in addressing therapy resistance in cancer. This novel understanding of cancer cell vulnerabilities not only elucidates the intricacies of therapy resistance but also paves the way for pioneering therapeutic opportunities, marking a critical step in the quest for more efficacious and tailored cancer treatments. By targeting tRNA modifiers alongside conventional therapies, we anticipate the development of highly effective and personalized treatment approaches.

## Mitochondrial tRNA modifications

### Mitochondrial tRNAs

Mitochondria are acknowledged as important organelles responsible for the majority of cellular production through oxidative phosphorylation (OXPHOS). They also play a crucial role in diverse cellular processes, including apoptosis, regulation of intracellular calcium levels, aging and involvement in metabolic pathways (154). In contrast to the nuclear DNA of eukaryotic cells, mitochondria house genetic material in the form of a multicopy, circular, double-stranded DNA encoding 13 polypeptides—the components of the OXPHOS system—along with two rRNAs and 22 tRNAs (155–157).

The 13 subunits synthesized by mitochondria assembled into the respiratory complex are essential for proper aerobic respiration and functional maintenance (158). In fact, dynamic gene expression adaptations and the regulation of intramitochondrial protein translation are important for adjusting to cellular energy demands and environmental conditions.

Within these processes and in the regulation of protein synthesis, tRNAs and their epitranscriptomic modifications are indispensable, with their deficiency significantly contributing to mitochondrial diseases (158,159). Most mitochondrial tRNAs (mt-tRNAs) assume non-canonical structures and undergo various modifications for stability and functionality (160). Unlike cytosolic tRNAs, mt-tRNAs adhere to a non-universal genetic code (161), allowing 22 mt-tRNAs, with 15 modifications at 118 positions, to encode 60 different codons (162). Despite the limited number of tRNAs in mitochondria, their modifications play diverse roles, underscoring their importance in efficient, accurate and dynamic protein synthesis (158).

**Figure 3.**
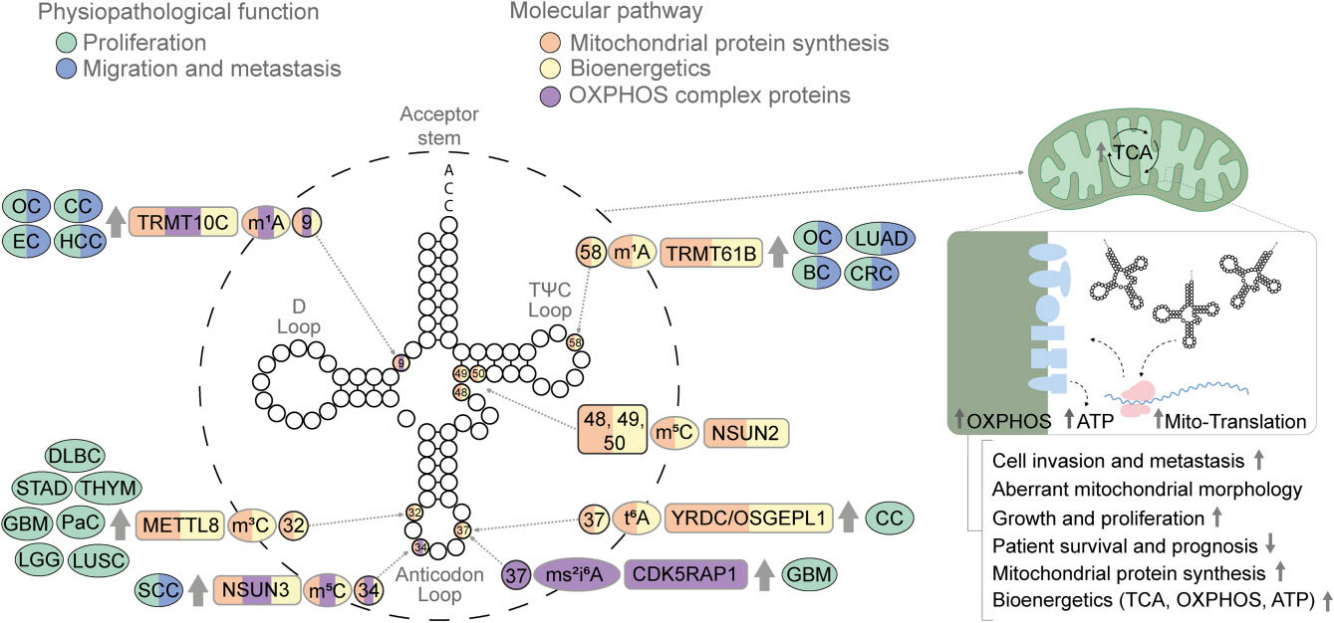
Mitochondrial tRNA modifications. The modifications and the corresponding writers are depicted in the tRNA backbone in different colors according to the oncogenic features in which they are involved, i.e. proliferation (green) and migration and metastasis (blue), as well as mitochondrial properties, i.e. mitochondrial protein synthesis (orange), altered bioenergetics (yellow) and OXPHOS activity (purple). The illustration progresses from the outermost to the innermost layers, providing details on the associated tumor types, the enzyme responsible for each mark and whether it is up- or down-regulated (indicated by arrows). The information includes the specific mark, along with the position where the enzyme catalyzes the mark. The category of solid tumors includes glioblastoma (GBM), endometrial carcinoma (EC), ovarian carcinoma (OC), cervical carcinoma (CC), lung adenocarcinoma (LUAD), colorectal cancer (CRC), breast cancer (BC), diffuse large B cell carcinoma (DLBC), low-grade glioma (LGG), lung squamous cell carcinoma (LUSC), pancreatic cancer (PaC), stomach adenocarcinoma (STAD), thyroid carcinoma (THYM) and squamous cell carcinoma (SCC).

Mitochondrial tRNA writers and erasers are encoded by nuclear DNA, translated into the cytoplasm and transported to the mitochondrial matrix, where they carry out their functions (157). Defects in these mitochondrial enzymes may contribute to diseases such as cancer (158) (Figure 3).

While the relationship between mt-tRNA modifications and cancer largely remains unknown, recent evidence suggests their significant role in maintaining bioenergetic balance and enabling tumor cells to adapt to the environment (163). In cancer-related mechanisms such as metastasis, mitochondria contribute to metabolic reprogramming, facilitating the dissemination of cells from the primary tumor (164). A more precise understanding of the mitochondria and cancer relationship could lead to therapies preventing tumor progression and dissemination, ultimately averting relapse.

Subsequently, in this section we will explore specific mt-tRNA modifications and tRNA-modifying proteins closely associated with mitochondrial protein synthesis and other characteristics, investigating their connections with cancer-related phenomena such as proliferation, survival and metastasis.

### Mitochondrial tRNA modifications

Within the NSUN family, NSUN2 localizes mainly to the cytosol, but it has been recently found localized in the mitochondria, directing C48, C49 and C50 methylation in the variable loop of mt-tRNA^Tyr^, mt-tRNA^His^, mt-tRNA^Leu^, mt-tRNA^Phe^, mt-tRNA^Glu^ and mt-tRNA^Ser^ (165,166). To investigate its implications in mitochondria, *NSUN2* KO HEK293T cells were engineered (165). Surprisingly, the absence of this modification only had a limited impact on mitochondrial translation and respiratory activity, showing no evident effects on the stability of mt-tRNAs and levels of OXPHOS complex proteins (165,166).

More recently, the role of the cytosine-5 mitochondrial methyltransferase NSUN3 has been implicated in cancer (164). NSUN3 is responsible for generating m^5^C at position 34 in mt-tRNA^Met^ (167,168). This modification plays a crucial role in the final deposition of the derivative 5-formylcytosine (f^5^C) by ALKBH1. Notably, a recent study by Delaunay *et al.* (164) unveiled a novel mechanism linking mt-tRNA modifications to the initiation of a reverse metabolic switch from glycolysis to OXPHOS, promoting tumor cell invasion and metastasis. In human oral cancer, higher *NSUN3* expression correlated with more advanced pathological states, and NSUN3 activity was particularly elevated near the tumor–stroma border, the site of invasion initiation (164). The study revealed that *NSUN3*-depleted SCC cell lines experienced a down-regulation of mitochondrial protein synthesis, a decrease in tricarboxylic acid metabolites and cellular respiration, reduced OXPHOS and an increase in glycolysis. Moreover, the down-regulation of *NSUN3* in head and neck cancer cells led to a reduction in lymph node metastasis in xenograft mice. Observing a relationship between NSUN3, development of metastasis and protein synthesis, the authors tried to block the prokaryotic protein synthesis machinery present in mitochondria with antibiotics that trigger components of the mitochondrial protein synthesis (linezolid, chloramphenicol, tigecycline and doxycycline), replicating the effects of down-regulating *NSUN3*, and resulting in reduced metastases both *in vitro* and *in vivo* (164). This intriguing study suggests that inhibition of mt-tRNA writers or mitochondrial protein synthesis could represent a promising therapeutic approach to impede the dissemination of tumor cells from primary tumors.

METTL8 catalyzes the production of 3-methylcytidine at position 32 in eukaryotic cytoplasmic tRNA^Thr^, tRNA^Ser^ and tRNA^Arg^ in both yeast and humans, and, more recently, it has been acknowledged in mt-tRNA^Thr^ and mt-tRNA^Ser^ species (169–172). *METTL8* is transcribed in a diverse array of variants produced through alternative splicing (173). One of the isoforms, isoform METTL8-Iso1, targets mitochondria through an N-terminal pre-sequence, thus preferentially modifying mt-tRNAs (174). *METTL8* KO cell lines with hypomodified mt-tRNAs exhibited slower growth and proliferation. Mechanistically, while aminoacylation and mitoribosome association remained unaffected, the translation of essential proteins in the energy transport chain was down-regulated, influencing OXPHOS capacity and mitochondrial bioenergetics (172,173,175). Research conducted by Schöller *et al.* (172), utilizing the TCGA database, found an up-regulation of *METTL8* in various cancers, including diffuse large B cell carcinoma (DLBC), GBM, low-grade glioma (LGG), lung squamous cell carcinoma (LUSC), pancreatic adenocarcinoma (PAAD), stomach adenocarcinoma (STAD) and thyroid carcinoma (THYM) (172). The study also revealed that in PAAD, elevated *METT8* levels correlated with lower patient survival and heightened respiratory chain activity (172). While the direct interconnection between increased METTL8-mediated mt-tRNA methylation and protein synthesis has not been explored in cancer, other isoforms of METTL8 have been shown to modify nucleolar RNAs, contributing to the formation of regulatory R-loops (176). In fact, this mechanism is crucial for the tumorigenicity of colon cancer cells in xenograft mouse models (176), suggesting a potential role for targeting METTL8 in therapeutic responses for cancer patients.

Overexpression of the RNA methyltransferase enzyme tRNA methyltransferase 61B (TRMT61B), resposible for the m^1^A58 of human mt-tRNAs, has been identified across a diverse array of cancer types in the TCGA tumor collection, and its expression correlates with the histopathological tumor grade in patients with CRC, BC, ovarian carcinoma (OC) and lung adenocarcinoma (LUAD) (177). Depletion of this writer leads to diminished cell proliferation and migration, accompanied by changes in mitochondrial morphology and reduced respiration, mitochondrial membrane potential and the protein expression of specific mitochondrial-encoded genes from the electron transport chain in melanoma cancer cell lines (177).

Another noteworthy yet less investigated writer is tRNA methyltransferase 10C (TRMT10C), responsible for methylating m^1^A at position 9 of mt-tRNAs (178). High expression of TRMT10C has been associated with a poor prognosis in cervical carcinoma (CC), endometrial carcinoma (EC), OC and HCC (179,180). Its oncogenic role has been substantiated through *in vitro* experiments involving *TRMT10C* KO ovarian and cervical cancer cells. These experiments revealed a significant reduction in proliferation, colony formation and migration (180). Interestingly, although its oncogenic role has not been directly linked to impaired mitochondrial protein translation, recessive mutations in *TRMT10C* in humans result in respiratory chain abnormalities, mitochondrial OXPHOS defects and a decrease in mitochondrial protein translation within the respiratory chain complex (178).

The mitochondrial methyl-thio-modifying enzyme, Cdk5 regulatory subunit-associated protein 1 (CDK5RAP1), plays a crucial role in maintaining the tumor-propagating capacity and self-renewal capacity of GSCs (181). CDK5RAP1 is responsible for converting *N*^6^-isopentenyladenosine (i^6^A) to 2-methylthio-*N*^6^-isopentenyl modification of adenosine (ms^2^i^6^A) at position A37 in mt-tRNAs, including mt-tRNA^Trp^, mt-tRNA^Tyr^, mt-tRNA^Phe^ and mt-tRNA^Ser^. While its deficiency does not affect mitochondrial respiration, it results in the loss of some components of the electron transport chain, influencing the metabolic phenotype of mitochondria and increasing autophagy. During gliomagenesis, cells leverage this mechanism to acquire a malignant phenotype (181).

Two other writers, YrdC *N*^6^-threonylcarbamoyltransferase domain containing (YRDC) and *O*-sialoglycoprotein endopeptidase like 1 (OSGEPL1), are responsible for the *N*^6^-threonylcarbamoyladenosine (t^6^A) modification at position 37 of mt-tRNAs, including mt-tRNA^Ser^, mt-tRNA^Thr^, mt-tRNA^Asn^, mt-tRNA^Ile^ and mt-tRNA^Lys^ (182,183). Mechanistically it is thought that the t^6^A modification has implications in various human diseases, including cancer, as its catalysis employs a high CO_2_/bicarbonate concentration, facilitating the development of solid tumors, which are predominantly found in hypoxic conditions (183). Its oncogenic role has been additionally shown by *OSGEPL1* depletion in HEK293T and HeLa cells. *OSGEPL1* KO cells exhibit reduced growth attributed to a lower oxygen consumption rate (OCR), and reduced protein levels of subunits of Complex I of the electron transport chain (183).

While still in its infancy, the comprehensive knowledge of mt-tRNA modifications unveils promising avenues for clinical interventions, especially in the realm of controlling tumorigenesis. This novel understanding of the modifications associated with mt-tRNA not only sheds light on the intricacies of tumorigenesis control but also opens the door to innovative therapeutic opportunities. By specifically targeting mt-tRNA modifiers, we envisage the development of highly effective and personalized treatment approaches for managing and controlling tumorigenesis.

## Conclusion and future directions

The perfect balance in tRNA modifications is essential for normal cell functions. Malfunction or an alteration in the tRNA-modifying enzymes responsible for tRNA modification deposition or removal might result in an abnormal phenotype, ultimately leading to tumor initiation. Thus far, numerous alterations in various tRNA-modifying proteins have been associated with different tumor types. These alterations contribute to tumor initiation, invasion, metastasis, immune escape and therapy resistance.

The increasing evidence for the contribution of alterations of RNA modifications to cancer may explain the boom in the field of epitranscriptomics. This field has rapidly evolved in the last few years. In just a decade, the field has been established and, 2 years ago, the first inhibitor targeting an RNA writer, METTL3, was introduced in clinical trials (184). This means that in less than 10 years of studying epitranscriptomics, it has been possible to develop a specific inhibitor, STM2457, against a writer that works efficiently, showing promising results in pre-clinical trials. Clinical trials for an METTL3 inhibitor began in November 2022 (identifier: NCT05584111). Furthermore, by 2023, a second-generation inhibitor of METTL3 was developed, demonstrating increased potency (185,186).

However, METTL3 is not the sole WER involved in m^6^A mRNA methylation for which a specific inhibitor has been developed. Some years ago, meclofenamic acid (MA2), an already in use and FDA-approved anti-inflammatory drug, was discovered to selectively inhibit FTO demethylase (187). This drug has undergone further studies in glioblastoma cells, where it impairs self-renewal (188) and enhances the antitumor effect of temozolomide (189). Promising results have also been observed in pre-clinical models of PCa (190). Clinical trials are already underway, testing its use in the meclofenamate form for GBM patients (191).

Since the discovery of the efficacy of the first inhibitor targeting METTL3, novel drugs aimed at epitranscriptomic targets have been developed to enhance treatments for cancer and various diseases. Notably, recent advancements include the creation of new molecules inhibiting NSUN6, NSUN2 and PUS7, with ongoing efforts in progress (97,192–194). In this era of big data, we now possess the opportunity to analyze physicochemical properties, molecular docking and compound library clustering through virtual screening. This approach allows us to identify compounds capable of inhibiting undruggable or challenging targets. Employing chemical space docking screens, NSUN2 and NSUN6 inhibitors have been identified as promising candidates (192,193).

The NSUN family members contain catalytic residues required for transferring the methyl groups to RNAs. In the case of NSUN2, two conserved active site cysteine residues (C271 and C321) have been identified in humans (195). Using these innovative predictive technics, an inhibitor for NSUN2 known as MY-1B was discovered (193). This compound is an azetidine acrylamide compound that acts as a ligand for C271 within the catalytic pocket of NSUN2. Another member of the NSUN family is NSUN6, which lacked previously designed inhibitors. However, employing diverse predictive techniques led to the design of initial compounds demonstrating affinities for NSUN6 (192). These compounds against NSUN family members are in early stages of development and need further efforts by the pharmaceutical industry to advance with the development of epitranscriptomic drugs as a new therapy for cancer treatment.

In parallel, new small molecule inhibitors are being virtually screened and predicted as promising candidates for modulating PUS7 activity and serving as a treatment against GBM (97,194). A pseudouridine isoxazolidinyl nucleoside analog was predicted using molecular modeling and *in silico* ADME-Tox profiling to effectively target PUS7 substrate. This compound exhibits moderate diffusion across cell membranes, permeates the blood–brain barrier and results in lower neurotoxicity (194). Another predicted small molecule demonstrates efficacy in suppressing glioblastoma tumorigenesis by reducing PUS7 activity in glioblastoma xenograft mouse models (97). These new-generation screening strategies represent a new era in the exploration of epitranscriptomic inhibitors, offering potential therapeutic candidates with higher efficiency and fewer adverse effects compared with conventional treatments for targeting cancers and other diseases.

Conventional treatments often fall short in yielding positive responses, frequently leading to the development of resistance or the onset of harmful adverse effects in many cases. This scenario is exemplified in treatments targeting mTOR. Over recent years, a variety of mTOR inhibitors have been developed and entered clinical trials for targeted therapy in tumors, organ transplantation, rheumatoid arthritis and other diseases (6). Despite efforts such as the use of paralogs, combinations of different mTOR inhibitors, ATP-competitive mTOR inhibitors, and second- and third-generation mTOR inhibitors, initial beneficial responses are commonly observed, but eventually tumors frequently develop resistance, leading to treatment failure (6,196). Furthermore, the inhibition of mTOR, an important signaling molecule for many pathways, has a cascade effect, inhibiting autophagy and increasing PI3K/Akt signaling, among other consequences, altogether boosting cancerous cell survival and metastasis. The interference with these vital cellular processes significantly diminishes the effectiveness of mTOR inhibitors in cancer treatment (197).

Hence, exploring novel therapeutic approaches targeting WERs offers potential avenues for reshaping the aberrant epitranscriptomic landscape within cancer cells, either by restoring normalcy or by hindering survival signaling pathways. These emerging strategies present exciting and promising possibilities for precisely eliminating cancer cells. The innovative screening strategies of this new generation could play an important role in advancing therapeutic interventions for RNA modifications. These interventions hold the promise of being safer, more effective, with reduced toxicities, and fewer adverse effects and recurrences compared with conventional treatments. We find ourselves at the dawn of an exciting new era in the realm of treatments, holding the prospect of significant advancements in the treatment and eradication of cancer.

## Data Availability

No new data were generated or analyzed in support of this research.
